# Light‐Ultrasound Driven Collective “Firework” Behavior of Nanomotors

**DOI:** 10.1002/advs.201800122

**Published:** 2018-05-02

**Authors:** Dekai Zhou, Yuan Gao, Junjie Yang, Yuguang C. Li, Guangbin Shao, Guangyu Zhang, Tianlong Li, Longqiu Li

**Affiliations:** ^1^ Key Laboratory of Microsystems and Microstructures Manufacturing Harbin Institute of Technology Harbin Heilongjiang 150001 China; ^2^ School of Mechatronics Engineering Harbin Institute of Technology Harbin 150001 China; ^3^ Department of Chemistry The Pennsylvania State University University Park PA 16802 USA

**Keywords:** aggregation, collective behavior, diffusion, firework behavior, nanomotors

## Abstract

It is of great interest and big challenge to control the collective behaviors of nanomotors to mimic the aggregation/separation behavior of biological systems. Here, a light‐acoustic combined method is proposed to control the aggregation/separation of artificial nanomotors. It is shown that nanomotors aggregate at the pressure node in acoustic field and afterward present a collective “firework” separation behavior induced by light irradiation. The collective behavior is found to be applicable for metallic materials and polymers even different light wavelengths are used. Physical insights on the collective firework behavior resulting from the change of acoustic streaming caused by optical force are provided. It is found that diffusion velocity and diffusion region of cluster can be controlled by adjusting light intensity and acoustic excitation voltage, and irradiation direction, respectively. This harmless, controllable, and widely applicable method provides new possibilities for groups of nanomachines, drug release, and cargo transport in nanomedicine and nanosensors.

## 1 Introduction

Nano/micromotors are nano/microscale devices^1^, ^2^, ^3^, ^4^, ^5^, ^6^, ^7^, ^8^, ^9^, ^10^, ^11^, ^12^ that can convert environmental energy, e.g., chemical,^13^, ^14^, ^15^, ^16^ magnetic,^17^, ^18^, ^19^, ^20^, ^21^, ^22^, ^23^ light,^24^, ^25^, ^26^ thermal,^27^, ^28^ electric,^29^, ^30^, ^31^ and acoustic^32^, ^33^, ^34^, ^35^ energies into mechanical energy. Wondrous biomimetic behaviors, including cargo transportation,^19^, ^36^ chemotaxis,^37^, ^38^ phototaxis,^39^, ^40^, ^41^ swarming,^42^, ^43^ and rheotaxis,^44^, ^45^ have been disclosed in the past decade. Recently, the swarming behaviors have attracted considerable attention^32^, ^46^, ^47^, ^48^, ^49^, ^50^, ^51^, ^52^, ^53^, ^54^, ^55^, ^56^, ^57^, ^58^, ^59^ since they can be used for fabricating materials with multifunctional properties (e.g., mechanical, conductive, and optical) and creating group of machines to achieve complex tasks.^60^, ^61^ Acoustic control is the fastest method to trigger the swarming behaviors of nano/micromotors in comparison to other sorts of energies.^61^, ^62^ Therefore, it is of great potential to be used for driving micromotors in biological applications, e.g., nanosurgery,^63^ biological targets,^64^ and siRNA delivery.^65^ As reported by Wang et al.,^32^ acoustic nanomotors can assemble into well‐ordered chains and aggregate on the pressure nodes. By using acoustic field, Xu et al.^58^ and Wang et al.^66^ achieved the swarming and separation behaviors of catalytic nanomotors. Other geometrical assembly behaviors of magnetic bimetallic nanomotors in acoustic combined magnetic field are reported by Ahmed et al.^67^ and Li et al.,^59^ respectively. These studies have further boosted out the application of acoustic propelled nanomotors. However, the chemical control method can only be used to control the collective behavior of bimetallic nanomotors, e.g., Au‐Pt, in a H_2_O_2_ environment. Moreover, the magnetic control method is only effective to the magnetic nano/micromotors. These limitations on solution and materials restrict their further applications in biological field. Therefore, a harmless and widely applicable method to control swarming behaviors of acoustic nanomotors is desirable. Compared with other types of energy, light energy is a form of direction controllable, power variable, and wireless energy which is capable of driving the motion of micro/nanomotors.^7^, ^68^, ^69^, ^70^ It shows great potential applications in the fields of biology and medicine. In this paper, we propose a light‐driven method to control the collective behavior of nano/micromotors. By applying a focused light on the swarming, a collective “firework” behavior of acoustic nanomotors can be obtained. The effects of light intensity, power of the acoustic field, nanomotors with different materials and solution types are presented. Depending on the experimental findings and numerical simulation results, a theoretical model is proposed to characterize the “firework” behavior. A complementary mechanism for the collective “firework” behavior is provided.^71^

## 2 Results and Discussion

**Figure 1:**
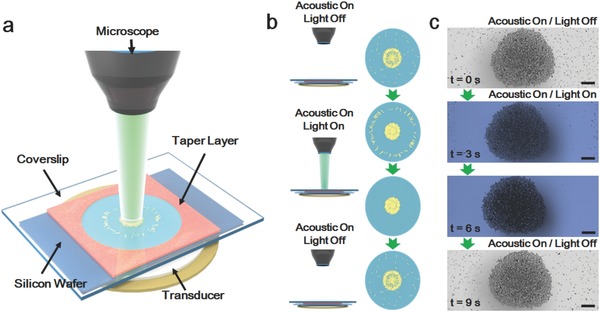
Schematic demonstration of experimental details and “firework” behaviors of light driven acoustic nanomotors. a) Schematic of the light combined acoustic cell for Au nanomotors. b) Schematic of the experimental processes. c) Time‐lapse snapshots of behaviors of Au naomotors. Excitation voltage, frequency, and light intensity are 10 V, 3 MHz, and 17 mW mm^−2^, respectively. Scale bar: 30 µm.

The experimental cell (**Figure** **1**a) was homemade that contained piezoelectric transducer as the ultrasonic source, silicon wafer, and tape layer as the container, coverslip as the acoustic reflector and the mercury lamp connected to the microscope as the light source. We first confirmed the motion behaviors of the Au nanomotors in an acoustic field without light irradiation. Details regarding to the synthetic procedures of the gold nanomotors and experimental procedures are provided in the Supporting Information. Schematic demonstration of experiment processes was presented in Figure 1b. When the acoustic frequency was ≈3 MHz and the applied voltage was 10 V, the Au nanomotors were levitated into the nodal plane by the ultrasound showing autonomous motion behaviors. In the meantime, the nanomotors swarm at the low‐pressure region to form a cluster. These behaviors are similar to the descriptions provided by Mallouk and co‐workers^66^ and Wangand co‐workers.^58^, ^59^ Subsequently, when the nanomotors cluster was formed in an acoustic field, a light (17 mW mm^−2^) irradiation was applied to the cluster, and hence, a collective outward firework‐like motion was observed for the Au nanomotors. The Au nanomotors on the edge or close proximity of the cluster start to move away from the core rapidly and form a vacant region. When the mercury lamp was turned off, the gold nanomotors move back, and recover its original cluster conformation. The time‐lapse snapshots of behaviors of Au nanomomotors are presented in Figure 1c. Experimental video is provided in Video S1 (Supporting Information).

**Figure 2:**
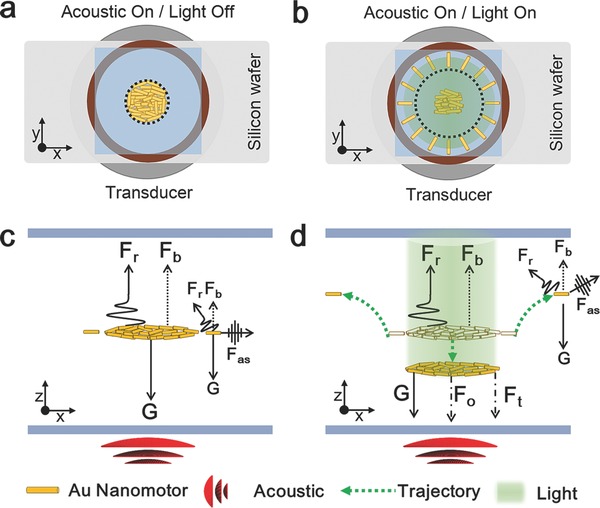
Illustration of forces balance. a) Schematic of Au nanomotors in acoustic field (Top view). b) Schematic of Au nanomotors in combined acoustic and light field (Top view). c) The force balance diagram on the cluster and Au nanomotors on the edge or close proximity of the cluster. d) The force balance diagram on the cluster and Au nanomotors with additional optical radiation force, *F*_o_.

As shown in **Figure** **2**a,b, Au nanomotors aggregate to form a cluster in acoustic field without light irradiation and the Au nanomotors on the edge or close proximity of the cluster diffuse away when they were irradiated. Aggregation behavior of Au nanomotors in acoustic field is well understood,^58^, ^59^, ^66^ i.e., when the acoustic field is turned on, there is a primary acoustic radiation force, *F*_r_, applied on the Au nanomotors in the vertical direction, as shown in Figure 2c. The radiation force can be calculated as^71^

(1) Fr= −πa3{2k03Ref1×p1× ∇p1−ρ0Ref2×v1× ∇v1

where *a* is the radius of particle, *k*_0_ and ρ_0_ are the compressibility and density of the working fluid, respectively; the dimensionless factors *f*_1_ and *f*_2_ (also called acoustic contrast factors) are calculated from the compressibility ratio and the density ratio between particle and fluid; *p*_1_ and *v*_1 _are the first‐order incident acoustic pressure and velocity field term, respectively, defined in the perturbation theory.^72^ The radiation force drives the nanomotors moving upward and finally levitates the Au nanomotors at the nodal plane due to the balance (Figure 2c) of the acoustic radiation force, *F*_r_, the gravitational force, *G*,  and the buoyancy force, *F*_b_, yielding

(2) $$G\;=F_{r}\;+\;F_{b}$$

An acoustic streaming around the cluster is formed as a result of the acoustic field and cluster, resulting in that the Au nanomotors on the edge or close proximity of the cluster are subjected to an additional acoustic streaming force, *F*_as_, which drives the nanomotors to assemble around the cluster as shown in Figure 2c.

When the mercury lamp is turned on, an extra optical radiation force, *F*_o_, is exerted on the cluster, which can be calculated as^73^

(3) $$F_{o}=\frac{P_{\mathrm{in}}}{c}\;\left(1+R\right)$$

where *P*_in_ is the energy transferred to a plane at a normal incidence angle per unit time and per unit area; *R* is the reflectivity of the nanomotors; *c* is the speed of light in vacuum. Due to the additional optical radiation force, the equilibrium of the forces on the cluster has been broken, yielding

(4) $$F_{o}+G\geq F_{r}+F_{b}$$

Thereafter, the nanomotors cluster move downward until the radiation force *F*_r_ is large enough to compensate and rebalance the optical radiation force *F*_o_. A new force diagram is shown in Figure 2d. As the cluster moves, the acoustic streaming around the cluster also changes. It indicates that the acoustic streaming force, *F*_as_, acted on the Au nanomotors at the edge or close proximity of the cluster, is different from before, and thereby, breaks the state of force balance. Thereafter, the Au nanomotors on the edge or close proximity of the cluster diffuse away as shown in Figure 2d.

**Figure 3:**
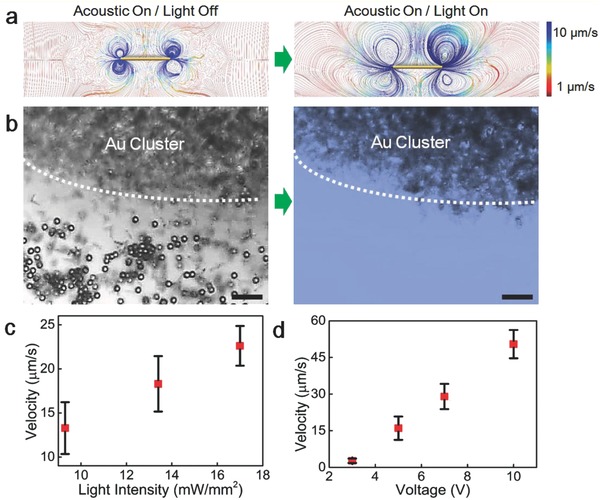
Streaming line around the cluster and the trajectory of tracers. a) Field vector for the acoustic streaming around nanomotor cluster without and with additional optical radiation force. b) “Firework” behaviors of tracer particles in acoustic field without and with light irradiation (with excitation voltage, frequency, and light intensity of 10 V, 3 MHz, and 17 mW mm^−2^, respectively), scale bar: 15 µm. c) Influence of light intensity on diffusion velocity of Au nanomotors (with excitation voltage of 6 V and frequency of 3 MHz). d) Influence of excitation voltage on diffusion velocity of Au nanomotors (with frequency of 3 MHz and light intensity of 17 mW mm^−2^).

In order to verify our hypothesis, numerical simulation on the acoustic streaming was carried out before and after light irradiation. Details on the simulation process are presented in the Supporting Information. As shown in **Figure** **3**a, the streaming line around the Au cluster was almost vertically symmetrical in acoustic field before light irradiation. Therefore, the direction of acoustic streaming force acted on the Au nanomotors at the edge or close proximity of the cluster was horizontal. When the light irradiation force was acted on, the Au cluster move downward until a new force balance is formed. Meanwhile, as the Au cluster moves away from the node plane, the vertical symmetry of streaming around the Au cluster is broken. Video on the change of streaming line before and after light irradiation was shown in Video S2 in the Supporting Information. It results in the direction change of acoustic force on the Au nanomotors. And finally, the nanomotors are pushed away from the cluster. To characterize the influence of streaming change, we added tracer particles in our simulation and calculated the trajectory of tracers before and after light irradiation. The trajectory of tracers confirmed our hypothesis (see Figure S6 and Video S3, Supporting Information). Finally, to examine the change of streaming, we added tracer particles (2 µm polystyrene microparticles with a negative zeta potential of −75 mV) in the solution. As shown in Figure 3b, the tracers aggregated around the Au cluster in the acoustic field without light irradiation. When the mercury light was turned on, these tracers diffuse away. These experimental results (see Video S4, Supporting Information) are consistent with our numerical simulation results. On the other hand, 2 µm amidine modified polystyrene microparticles with a positive zeta potential of 33 mV were also used as tracers. As we found, the tracers with negative or positive zeta potential were pushed away from the middle under the same light irradiation. See Video S4 in the Supporting Information. It suggests that the motion behaviors are not induced by a light‐generated electric field. Otherwise, the microparticles with different zeta potentials should present different diffusion direction.^50^ Furthermore, we have examined the “firework” motion in salt solutions. Similar “firework” behaviors were observed for Au nanomotors in a 0.05 m NaCl solution, see Video S5 in the Supporting Information. We could confirm that the salt concentration has no effect on the diffusion behaviors. According to above discussion, we can eliminate the photoelectric effect which may generate electric field or induce electrophoresis to form a diffusion pattern similar to the “firework” motions.^54^ From what we observed in experiment and numerical simulation, the effect of light intensity and acoustic excitation power, should be investigated. We first measured the influence of light intensity on the diffusion speed of Au nanomotors, while holding the acoustic field constant. As shown in Figure 3c, variation in light intensity from 8 to 17 mW mm^−2^ were achieved by applying different neutral density filters (ND 0.1 and ND 0.3). Hence, the diffusion velocities increase with increasing light intensity. Thus, light plays a significant role in the collective behaviors of Au nanomotors. The motions videos of nanomotors under different light intensities are available in Video S6 (Supporting Information). Second, we investigated the influence of the acoustic excitation voltage on the diffusion speeds for the same light intensity (17 mW mm^−2^) and frequency (3 MHz). As can be seen from Figure 3d, the relationship between excitation voltage and diffusion velocity is a linear correlation. When the excitation voltage is 10 V, the diffusion velocity can reach up to 50 µm s^−1^. As the excitation voltage decreases to 3 V, the nanomotors are insensitive to light irradiation, i.e., there is no obvious “firework” behavior observed (Video S7, Supporting Information). It indicates that both of the acoustic and light field are indispensable. If the “firework” phenomenon was induced by light‐thermal effect,^68^ only light irradiation is required and hence, acoustic excitation is not necessary, which is inconsistent with what was observed in our experiments. Meanwhile, through the numerical simulation, we found that the streaming induced by the light‐thermal was really tiny and could not induce such a drastic “firework” behavior (Figures S7–S9, Supporting Information). Thus, the light‐thermal effect is not a key factor that generates the firework motions in our experiments.

To further explore the “firework” behavior and expand its potential applications, different materials of nanomotors, e.g., single metallic materials (Pd, Ag, as shown in Figure S1a,b, Supporting Information), metal‐metalloid core‐shell materials (Au coated SiO_2_, as shown in Figure S1c, Supporting Information), metalloid materials (SiO_2_ as shown in Figure S1d, Supporting Information) and polymer (Polypyrrole as shown in Figure S1e, Supporting Information) were synthesized to examine their motion behaviors in acoustic combined light field. These materials of nanorods are widely used in different areas, e.g., sensors, catalyst, solar cell.^74^, ^75^, ^76^ Details related to the sample preparations are described in the Supporting Information and the motion videos of different nanomotors are provided in Video S8 (Supporting Information).

**Figure 4:**
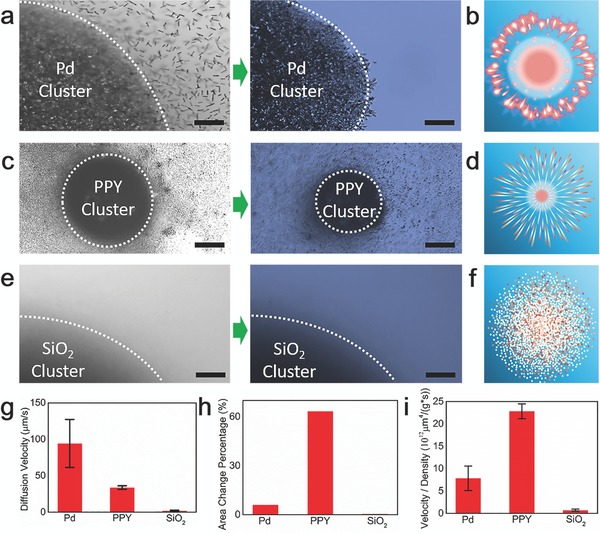
Varied “firework” behaviors of nanomotors prepared with different materials. a,c,e) Aggregation of Ag, PPY, and SiO_2_ nanomotors before and after light irradiation in acoustic field. Excitation voltage, frequency, and light intensity are 10 V, 3 MHz, and 17 mW mm^−2^, respectively. Scale: bar 20 µm. b,d,f) Illustration of “firework” behavior of Pd, PPY, and SiO_2_ nanomotors in combined acoustic and light field. g) Diffusion velocity of different nanomotors with light irradiation in acoustic field. h) Area change percentage of different nanomotors cluster before and after light irradiation in acoustic field. i) Ratio of diffusion velocity and density of nanomotors with different materials.

As shown in **Figure** **4**a, the metallic nanomotors (Pd) show similar “firework” behavior with Au nanomotors, i.e., in acoustic field, Pd nanomotors aggregate to form a cluster and the Pd nanomotors on the edge or close proximity of the cluster diffuse away caused by light irradiation (Figure 4b). It should be noted that the diffusion behavior of the Au coated SiO_2_ nanomotors is similar to that of Au nanomotors without SiO_2_. This finding suggests that the “firework” behavior is not induced by the surface reaction of the nanomotors since the metallic surface has been covered by SiO_2_. We note from Figure 4c that the diffusion behaviors of PPY nanomotors are much more responsive than the Au nanomotors. The PPY nanomotors rapidly diffuse outward and form a small core of cluster once the light irradiation was turned on (Figure 4d). However, there is no remarkable diffusion motion for the pure SiO_2_ nanomotors, as shown in Figure 4e,f. This is because SiO_2_ is a light transmitting material. Comparing the results between Au, PPY, and SiO_2_, the diffusion velocity of Pd nanomotors is the fastest (see Figure 4g). However, due to the difference in density, the acoustic radiation force (see Equation (1)) on Pd is much larger than that on the other two materials which enhance the difficulty to diffuse away from the cluster.

As presented in Figure 4h, the area change perchance of Pd nanomotors cluster is about 6% which is ten times smaller than PPY nanomotors clusters and the ratio of diffusion velocity and density of PPY nanomotors is the highest as shown in Figure 4i. On the other hand, as the SiO_2_ is a light transmitting materials, the optical force acting on the SiO_2_ nanomotors cluster was almost zero. Therefore, there is no remarkable change for the diffusion velocity and area for the SiO_2_ cluster. From the above, we can confirm that the transmission and density of nanomotors are key factors that could influent the “firework” diffusion velocity.

**Figure 5:**
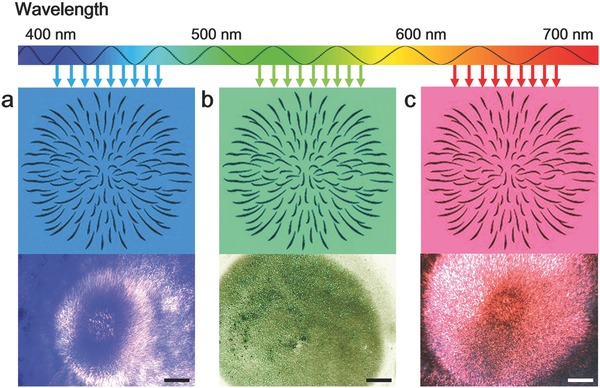
Lights driven collective “firework” of PPY nanomotors for different lasers. a) Blue laser b) Green laser c) Red laser. All experiments were performed under 10 mW mm^−2^ light intensity, 10 V excitation voltage, and 3 MHz frequency. Scale bar: 20 µm.

Since the PPY nanomotors presented excellent optical response characteristics, we kept exploring their response to the wavelength of light. As shown in **Figure** **5**a–c, lasers with different wavelength (blue, green, red) were applied on the PPY nanomotors cluster. As we found, the PPY nanomotors show similar explosive “firework” behaviors even different wavelength lasers were used (Video S9, Supporting Information).

**Figure 6:**
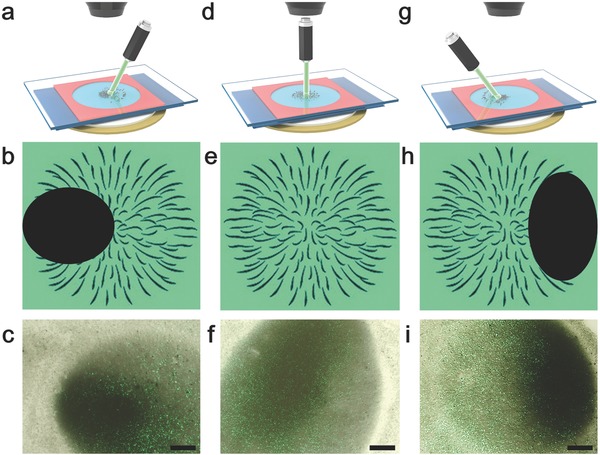
Controlling diffusion position of PPY nanomotors cluster by altering the light direction. a,d,g) Schematic of light irradiation on PPY nanomotors cluster at different direction (top right, top, top left). b,e,h) Illustration of “firework” behaviors of PPY nanomotors cluster at different position in acoustic field. c,f,i) Experimental results of “firework” behavior of PPY nanomotors cluster at different position in acoustic field. Excitation voltage, frequency and light intensity were 10 V, 3 MHz, and 17 mW mm^−2^, respectively. Scale bar: 20 µm.

Finally, we demonstrate shape control for the “firework” behaviors of PPY nanomotors. As shown in **Figure** **6**a,d,g, green laser was applied on the PPY cluster at different direction (top right, top, and top left). When the PPY aggregation was irradiated at a tilted angle by laser, only part of the nanomotors cluster which was irradiated diffuse away, i.e., the other part remains stationary as shown in Figure 6b,e,h. Experimental results were shown in Figure 6c,f,i). See Video S10 in the Supporting Information. It indicates that the “firework” diffusion can be controlled by changing the irradiation position and angles. Those optical responses are useful for the applications in controlled drug release and nanosensors.

## 3 Conclusion

In conclusion, we have demonstrated the ability of light field to control the aggregation/diffusion behavior of acoustic nanomotors. In particular, a collective “firework” behavior of acoustically driven nanomotors are presented by applying an additional light irradiation. Light intensity, excitation voltage, transmission, and density of materials are found to be the key factors affecting diffusion velocity. Experimental findings and numerical simulation results confirmed that this behavior was triggered by acoustic streaming resulting from optical force of light irradiation. Meanwhile, typical lights with different wavelengths of provide similar collective “firework” behaviors. The diffusion region of the cluster can be controlled by changing irradiation direction. The light driven collective “firework” behavior of acoustic nanomotors with the advantages of reversible, wireless, and controllable on demand explores the applications in biological field, ranging from chemical sensing to nanomachinery and drug delivery.

## Conflict of Interest

The authors declare no conflict of interest.

## Supporting information

Supplementary

Supplementary

Supplementary

Supplementary

Supplementary

Supplementary

Supplementary

Supplementary

Supplementary

Supplementary

Supplementary
