# Supplementary material for: Light‐Ultrasound Driven Collective “Firework” Behavior of Nanomotors
Source: Adv Sci (Weinh). 2018 May 2;5(7):1800122. doi: 10.1002/advs.201800122 (PMC6051403; doi:10.1002/advs.201800122)
Supplement: Supplementary file 1 — Supplementary [file ADVS-5-1800122-s002.pdf]

Copyright WILEY-VCH Verlag GmbH & Co. KGaA, 69469 Weinheim, Germany, 2018.

ADVANCED SCIENCE
Open Access

Supporting Information

for *Adv. Sci.,* DOI: 10.1002/advs.201800122

Light-Ultrasound Driven Collective "Firework" Behavior of Nanomotors

Dekai Zhou, Yuan Gao, Junjie Yang, Yuguang C. Li, Guangbin Shao, Guangyu Zhang, Tianlong Li, and Longqiu Li**

Copyright WILEY-VCH Verlag GmbH & Co. KGaA, 69469 Weinheim, Germany, 2016.

Supporting Information

Light-ultrasound Driven Collective "Firework" Behavior of Nanomotors

Dekai Zhou, Yuan Gao, Junjie Yang, Yuguang C. Li, Guangbin Shao, Guangyu Zhang, Tianlong Li, and Longqiu Li**

Supporting videos

Video S1: Light driven collective "Firework" behavior of Au nanomotors in acoustic field (excitation voltage 10 V, frequency 3 MHz and light intensity 17 mW mm^{-2})

Video S2: Change of streaming line before and after light irradiation through numerical simulation.

Video S3: Numerical simulation of tracers trajectory around Au cluster without and with light irradiation in acoustic field.

Video S4: Diffusion behavior of polystyrene microparticles with negative or positive zeta potential. Excitation voltage, frequency and light intensity was 10 V, 3 MHz and 17 mW mm^{-2}, respectively.

Video S5: Diffusion behavior of tracer particles in 0.05 M NaCl solution. Excitation voltage, frequency and light intensity was 10 V, 3 MHz and 17 mW mm^{-2}, respectively.

Video S6: Diffusion velocities of Au nanomotors at different light intensities with same excitation voltage (6 V) and frequency (3 MHz).

Video S7: Diffusion velocities of Au nanomotors at different excitation voltages with light intensity of 17 mW mm^{-2}.

Video S8: "Firework" behaviors of nanomotors with different materials. Excitation voltage, frequency and light intensity was 10 V, 3 MHz and 17 mW mm^{-2}, respectively.

Video S9: Different colors response of PPY nanomotors cluster. Excitation voltage, frequency and light intensity was 10 V, 3 MHz and 10 mW mm^{-2}, respectively.

Video S10: Shape control for the “firework” behaviors of PPY nanomotors. Excitation voltage, frequency and light intensity was 10 V, 3 MHz and 10 mW mm^{-2}, respectively.

1. Synthesis of acoustic nano-/micromotor

Metallic (Au, Pd, Ag) and polypyrrole (PPY) nanorods were synthesized using anodic alumina membranes (AAO, Whatman Inc.) by electrodepsotion method. Note that the pores are uniformly distributed among the membrane and the diameter of the pores is 200 nm. The metal plating solutions for single metallic materials (Au, Ag, Pd) and copper etching solutions were purchased from Technic Inc., and these solutions were used without any further purification. Prior to deposition of nanorods, Cu film was deposited on the back side of AAO membranes using a Kurt Lesker Lab-18 electron beam evaporator. The copper covered AAO membranes and Pt wire serve as working and counter electrodes in the electrochemical cell, respectively. For the deposition of Au, Pd, Ag, and Cu, Pt coil was employed as pseudoreference electrode, whilst a saturated Ag/AgCl electrode was introduced as genuine reference electrode in the system. Au, Pd, Ag and Cu nanorods were fabricated in constant current density mode, and the current strength were set into -1.24, -2.12, -1.77, 1.42 mA cm^{-2} respectively, while the deposition of polypyrrole nanorods was conducted in constant potential mode of 0.7 V. In atypical deposition procedure, *e.g.* Au nanorods, the fabrication of nanorods normally initializes from the deposition of Cu composition to fill the sacrificial layer. After that, the chamber was cleaned with deionized (DI) water and then filled with the gold plating solution. After the electrodeposition of gold, the AAO membrane was rinsed with DI water and dried. The copper layer was dissolved using the copper etching solution and 3 M NaOH was used to dissolve the AAO membrane to release the gold nanorods. In final, the Au nanorods were washed in DI water several times for further experiments. SiO_2 coated Au

nanowires were synthesized based on previous report.[1] Briefly, nanowires from a whole membrane were suspended in 1 mL of ethanol. Then, 300 μL Au nanowires, 490 μL of ethanol, 160 μL of distilled water, 40 μL of tetraethylorthosilane and 10 μL of 28% NH_4OH were mixed and sonicated for 45 min. After the sonication, the solution were washed three times with ethanol and released in DI water for further applications. Silica microrods were prepared based on previous report.[2] Briefly, in a 500 ml bottle, 30 g of PVP (average molecular weight Mn = 40.000, Sigma-Aldrich) was dissolved in 300 ml of 1-pentanol (⩾ 99%, Sigma-Aldrich) and sonicated for 2 hours. When all PVP had been dissolved, 30 mL ethanol, 8.4 mL DI water and 2 ml of 0.18 м sodium citrate dihydrate (99%, Sigma) solution in water was added to the pentanol. The flask was shaken by hand to mix the content. Then, 6.75 ml of ammonia (25 mass percent in water, Merck) was added, the flask was shaken again and 3 ml of TEOS (⩾ 98%, Fluka) was added to the mixture. After shaking again, the bottle was left to rest and the reaction was allowed to proceed overnight. Next, the reaction mixture was centrifuged for 1 hour. These microrods was cleaned with ethanol, water two times, respectively.

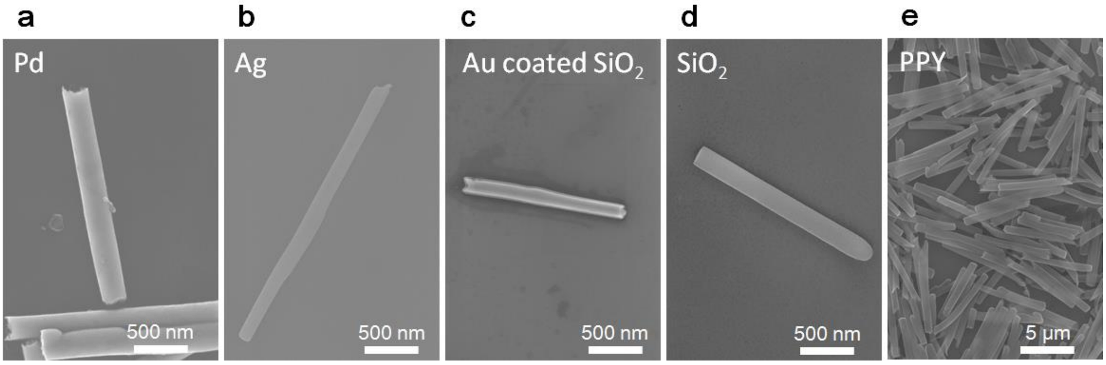

Figure S1. Scanning electron microscope images of different nanorods. (a) Pd. (b) Ag. (c) Au coated SiO_2. (d) SiO_2. (e) PPY.

2. Acoustic combined with light experiments.

A piezoelectric ceramics piece was pasted on the bottom of silicon wafer, then three layers of polyimide Kapton tape (60 um thickness per layer) with a circular hole of 6 mm diameter were on the top of the silicon wafer to serve as the chamber. After that, 30 μL of nanorods dispersion was dropped into the cell and covered by a square glass coverslip which was served as the sound reflector. A signal generator (5062 Tabor Electronics, Israel) was connected to the piezoelectric ceramics piece to serve as signal source. Mercury light connected with the microscope objective was used as light source. The variation of light intensity was adjusted by adjusting neutral density filters.

3. Video recording and tracking

The motion of nanomotors were observed by an Olympus BX60M optical microscope and recorded by a video capture device (Dazzle Video Creator Plus). Videos of the nanomotors were captured at 30 frames per second. The motion of nanomotors was analyzed by PhysMo software (PhysMo - Video Motion Analysis Package, http://physmo.sf.net). The average speeds of different nanomotors were determined by calculating the mean values of at least ten different samples.

4. Material characterization

The morphology of nanowires were characterized by using a FEI NanoSEM 630 scanning electron microscope. UV-vis diffuse-reflectance spectra of these nanowires on glass slides were measured by using Perkin-Elmer Lambda 950 spectrophotometer. The zeta potential of the microspheres was measured by a Malvern instruments Zen 3600 Zetasizer.

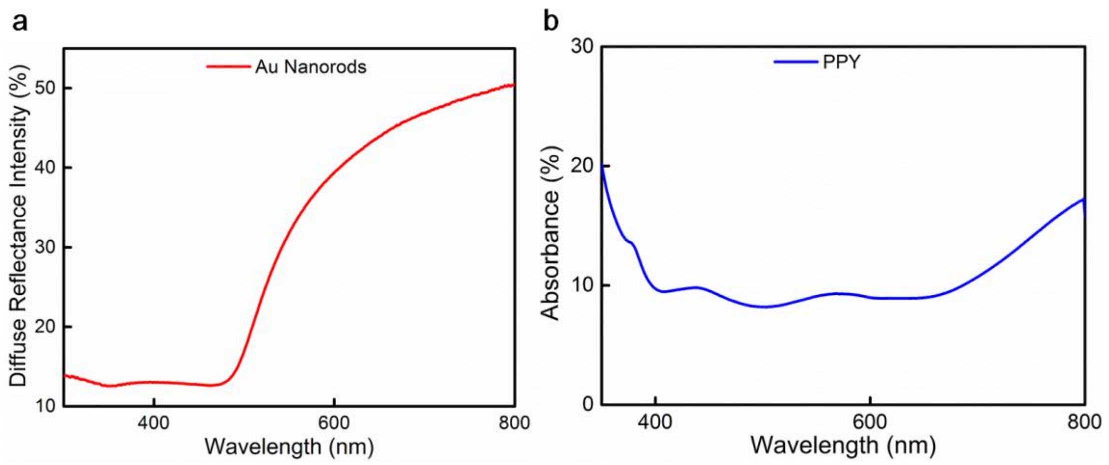

Figure S2. (a) Diffuse reflectance spectrum of the Au nanorods on a glass slide. (b) Absorption spectrum of the PPY film on a glass slide.

5. Simulation of the acoustic streaming without light irradiation.

Commercial finite element analysis software, COMSOL Multiphysics, was employed to simulate the collective "firework" behavior of fabricated nanorods. Thermal viscous acoustic and fluid-solid modules were coupled to study the acoustic streaming before and after applying light on the cluster. Moreover, particle tracing module was further integrated into current model for tracing the trajectory of the particles around the gold cluster. As shown in **Figure S3**, the cross-section of the whole cell was converted into a two-dimensional (2D) model, in which the cluster was surrounded by a rectangular water medium at the geometric center. The width and length of the cell were 1000 μm and 250 μm (half of wave length), respectively, while dimension of gold nanorods cluster was set into 100 × 5 μm corresponding to the experimental results. Any axial dynamics and boundary streaming induced by the wave reflectance by hitting the walls were neglected in our model since the size of Au cluster was fairly small enough in comparison to that of the whole cell. [3] Thus, only one pressure nodal (variable) was available in 2D field.

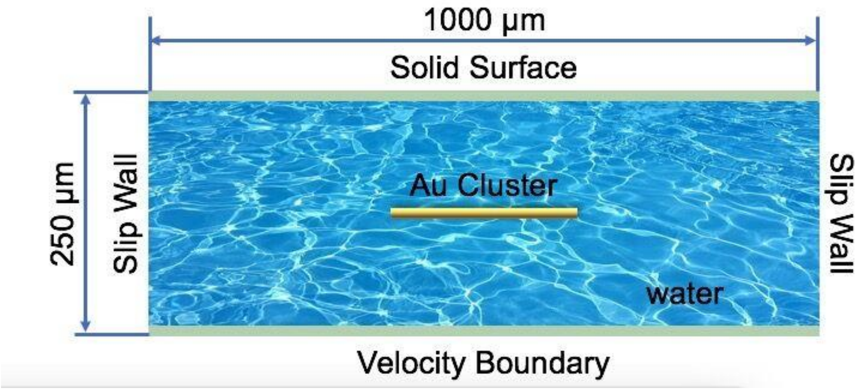

Figure S3. Illustration of 2D configuration of the simulation model.

In general, acoustic streaming comprises Eckart streaming and boundary streaming. However, in current model, the Eckart streaming effect is significantly low in standing waves, which is not considered during the calculation.[4] Therefore, acoustic perturbations of the first and second order was simulated by perturbation theory, which can be expressed as [5-7]

$$T = T_0 + T_1 + T_2 \tag{1}$$

$$p = p_0 + p_1 + p \tag{2}$$

$$v = v_1 + v_2 \tag{3}$$

The ultrasound actuation was applied through boundary conditions on the first-order velocity v_1 and temperature T_0.

Besides, two thermodynamic relations (Equation 4 and Equation 5) are used to describe the changes in ρ and s

$$d\rho = \gamma\kappa\rho d\rho - \alpha\rho dT \tag{4}$$

$$ds = \frac{C_p}{T}dT - \frac{\alpha}{\rho}dp \tag{5}$$

where C_p is the specific heat capacity at constant pressure; γ is the specific heat capacity; κ is the isentropic compressibility and α is the isobaric thermal expansion coefficient. These parameters are given by

$$\gamma = \frac{C_p}{C_v} \tag{6}$$

$$\kappa = \frac{1}{\rho}(\frac{\partial\rho}{\partial P})_s \tag{7}$$

$$\alpha = -\frac{1}{\rho}(\frac{\partial\rho}{\partial T})_p \tag{8}$$

The thermal viscous module in the first order equations are expressed in terms of T_1, p_1 and v_1,[8]

$$\partial_t T_1 = D_{th}\nabla^2 T_1 + \frac{\alpha T_0}{\rho_0 C_p}\partial_t p_1 \tag{9}$$

$$\partial_t p_1 = \frac{1}{\gamma\kappa}[\alpha\partial_t T_1 - \nabla\cdot v_1] \tag{10}$$

$$\rho_0\partial_t v_1 = -\nabla p_1 + \eta\nabla^2 v_1 + \beta\eta\nabla(\nabla\cdot v_1) \tag{11}$$

where D_{th} is the thermal diffusivity; η is the dynamic viscosity and β is the viscosity ratio.

Assuming that the first order terms in Equations 9-11 have a harmonic time dependence e^{-iwt}, then by using the thermodynamic identity equation (Equation 12)

$$T_0\alpha^2/\rho_0 C_p\kappa = \gamma - 1 \tag{12}$$

Equations 9-11 can be simplified as follow [9]

$$i\omega T_1 + \gamma D_{th}\nabla^2 T_1 = \frac{\gamma-1}{\alpha}\nabla\cdot v_1 \tag{13}$$

$$i\omega v_1 + \upsilon\nabla^2 v_1 + \upsilon\left[\beta + i\frac{1}{\gamma\rho_0\kappa\upsilon\omega}\right]\nabla(\nabla\cdot v_1) = \frac{\alpha}{\gamma\rho_0\kappa}\nabla T_1 \tag{14}$$

$$\upsilon = \eta/\rho_0 \tag{15}$$

From Equations 13, 14 and 15, thermal and viscous penetration depth can be deduced

$$\delta_{th} = \sqrt{2D_{th}/\omega} \tag{16}$$

$$\delta = \sqrt{2\upsilon/\omega} \tag{17}$$

Since the ultrasound frequency used in the experiment is 3 MHz, δ_{th} = 0.13µm and δ = 0.31 µm, these values were carefully considered when specifying the length scale of boundary layers in the fluid-solid boundary and maximum and minimum element size length.

As for the second order, continuity equation and Navier–Stokes equation are

$$\partial_t\rho_2 = -\rho_0\nabla\cdot v_2 - \nabla\cdot(\rho_1 v_1) \tag{18}$$

$$\rho_0\partial_t v_2 = -\nabla p_2 + \eta\nabla^2 v_2 + \beta\eta\nabla(\nabla\cdot v_2) - \rho_1\partial_t v_1 - \rho_0(v_1\cdot\nabla)v_1 \tag{19}$$

Since there is no necessary to consider the microsecond timescale, the time-averaged second-order equations[10] are

$$\rho_0 \nabla \cdot < v_2 > = -\nabla \cdot < \rho_1 v_1 > \quad (20)$$

$$\eta \nabla^2 < v_2 > + \beta\eta\nabla(\nabla \cdot < v_2 >) - < \nabla p_2 > = < \rho_1 \partial_t v_1 > + \rho_0 < (v_1 \cdot \nabla) v_1 > \quad (21)$$

The acoustic streaming was introduced by adding the volume force and the mass source (weak contribution) through the fluid-solid interaction module, i.e., the first order terms were the source of second order terms.[4]

The obtained results are shown in **Figure S4**, gold cluster is levitated by radiation force at the nodal plane and four bull streaming rolls are clearly observed around the nanorods cluster, which indicates that the streaming is induced by the boundaries of gold cluster. Moreover, the streaming line around the Au cluster is almost vertically symmetrical in acoustic field.

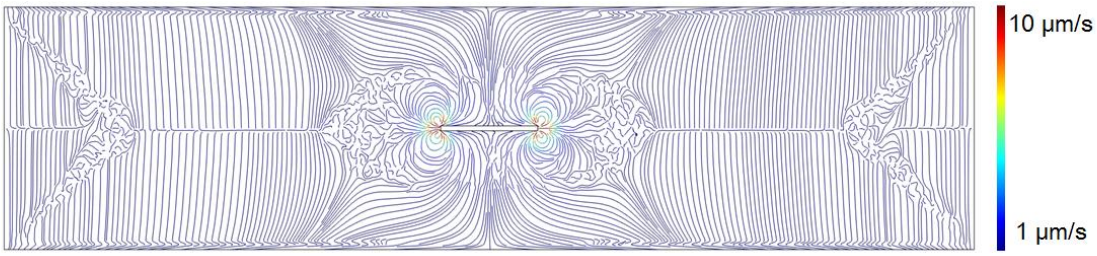

Figure S4. Acoustic streaming around the cluster. Streaming rolls of which velocity is lower than 1×10^{-7} m s^{-1} are neglected.

6. Simulation of light irradiation force for acoustic streaming flow

In order to analyze the collective behavior of cluster and particles around the cluster, fluid-solid coupling module and particle-tracing module were combined to investigate the streamline and track the movement of fluid, separately. Before being irradiated by light, gold cluster is subjected to acoustic radiation force[8], stokes drag force, gravity and buoyancy force, yielding

$$F_r = -\pi a^3 \{\frac{1\kappa_0}{3} Re[f_1 \times p_1 \times \nabla p_1] - \rho_0 Re[f_2 \times v_1 \times \nabla v_1]\} \quad (22)$$

$$F_{drag} = -n \cdot (-pI + \eta(\nabla v_1 + (\nabla v_1)^T)) \quad (23)$$

$$G = \frac{4}{3}\pi a^3(\rho - \rho_0)g \quad (24)$$

where a is the radius of particle; κ_0 is the compressibility of fluid; p is the pressure of the fluid. The dimensionless factors f_1 and f_2 (also called acoustic contrast factors) are calculated from the compressibility ratio and density ratio between the particle and fluid. ρ_0 is the density of the fluid; p_1 and v_1 are the first-order terms defined in the perturbation theory; η is the dynamic viscosity of the fluid. After light irradiation, there was an additional force, optical force in the -z direction[11]

$$F_o = P_{in} \times (1 + R)/c \quad (25)$$

where P_{in} is the energy transferred to a plane at normal incidence per unit time and per unit area; R is the reflectivity (calculated from Figure S2); c is the speed of light.

This additional force caused the downward motion of the cluster and changes the streaming pattern around the cluster, and subsequently, changes the alignment of the particles around. The process is simulated by the time dependent study method through COMSOL fluid-solid interaction module. Mesh convergence was checked to ensure the mesh quality throughout simulation. The streaming pattern after exerting irradiation is shown in **Figure S5.** As the Au cluster left the node plane, the vertical symmetry of streaming around the Au cluster was broken.

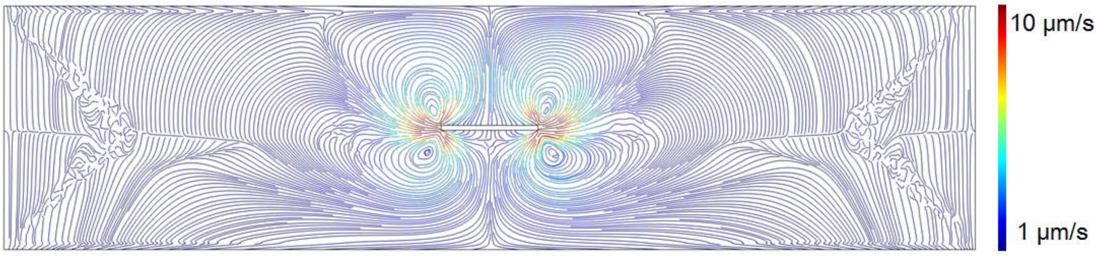

Figure S5. Acoustic streaming around the cluster after light irradiation. Streaming rolls of which velocity is lower than 1×10^{-7} m s^{-1} are neglected.

The particle tracing results provided a more intuitive view for the alignment of particles and ease the comparison between simulation and experiments. The particle alignment in the whole process is shown in **Figure S6**. Before light irradiation, particles begin to aggregate at the nodal plane as shown in Figure S6 (a-c) due to the acoustic radiation force. When the mercury light is turned on, an extra optical radiation force is exerted on the cluster, which resulted in the change of acoustic streaming around the cluster as shown in Figure S5. Hence, the acoustic streaming force acting on the tracer particles changes and the tracer particles on the edge or close proximity of the cluster begin to diffuse away from the Au cluster as shown in Figure S6 (d-e).

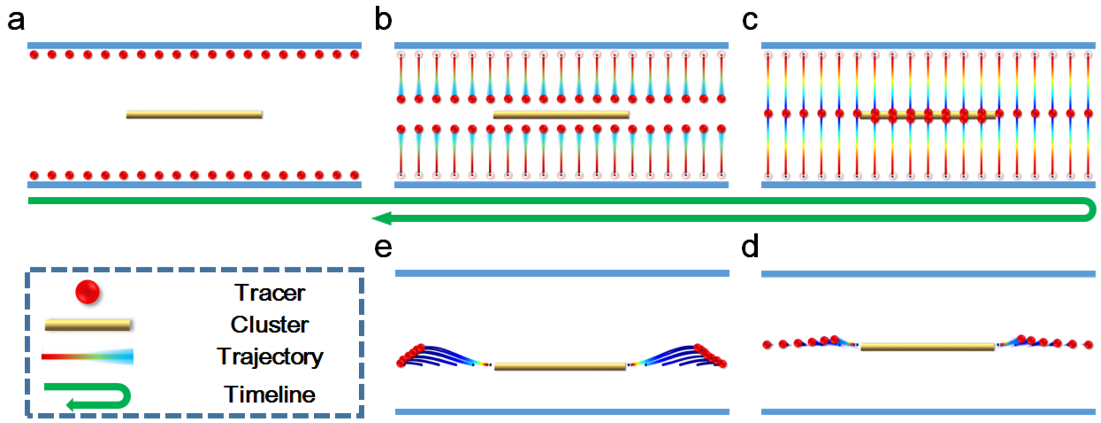

Figure S6. Particle alignment of the whole process. (a) Particles released with no velocity. (b - c) Particles assembled at a pressure nodal plane. (d - e) Particles diffused away from the aggregation.

7. Simulation of the temperature distribution with light irradiation

Two models were built for investigating the influence of thermal effect on the diffusion behavior of the cluster after light irradiation. Based on the light-thermal effect, a non-isothermal flow would generate in the cell. According to the Fourier's heat conduction law, the irradiation process in current scale can be analyzed in macro-scale.[2] The size effect can be neglected in the simulation process.

Considering our experimental results, the "firework" behavior still existed after 16 s. As shown in Figure S7, the stationary result can be mathematically expressed as

$$\rho C_p u \cdot \nabla T + \nabla \cdot q = Q \tag{26}$$

where ρ is the density; u is the velocity vector of the translational motion; q is the heat flux. The final temperature distribution was shown in **Figure S7**.

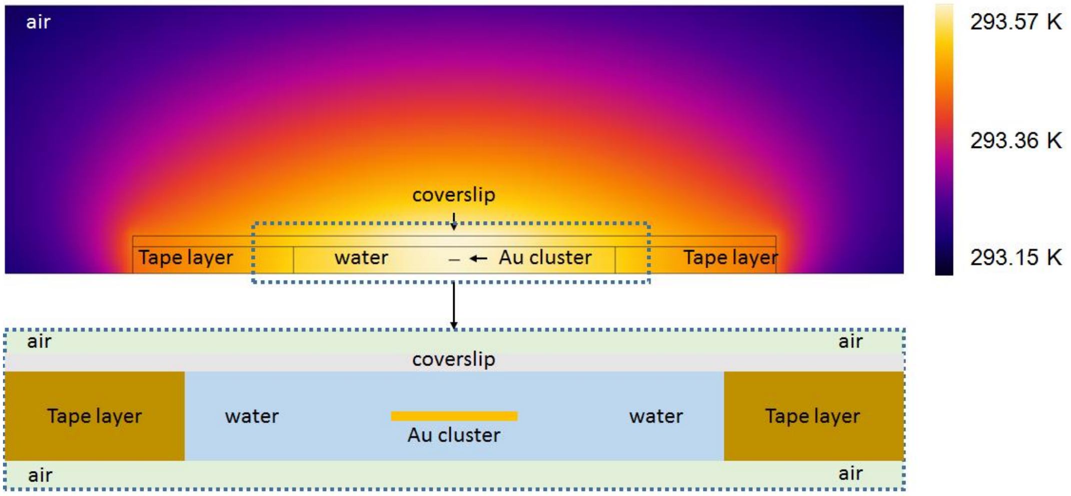

Figure S7. Temperature distribution in the two-dimensional configuration model under light irradiation.

8. Simulation of thermal induced acoustic streaming flow

Irradiated by light, the fluid properties could be changed with the rise of temperature. As interpreted by Equation 20, the material properties that relate to the amplitude of acoustic streaming were all denoted in the interpolation function including density ρ, heat capacity C_p and compressibility κ. Therefore, temperature distribution was further implemented into acoustic streaming model that study the variations of acoustic streaming caused by the rise of temperature.

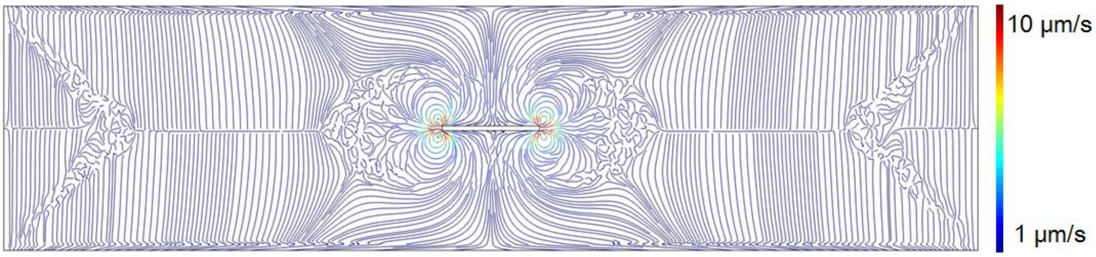

Figure S8. Acoustic streaming line variation induced by fluid properties change due to light-thermal effect.

As shown in **Figure S8**, the amplitude of acoustic streaming imposed negligible variations before and after irradiation, i.e., the "firework" behavior is not related to the change of material properties induced by the rise of temperature.

9. Simulation of thermal gradient induced flow

In order to obtain the influence of light-thermal effect induced non-isothermal flow on the diffusion behaviors, heat transfer module and laminar flow module are used to calculate the strength of the non-isothermal flow.

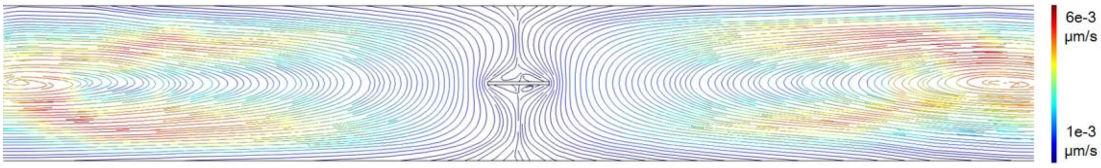

Figure S9. Streaming line of the non-isothermal flow.

As shown in **Figure S9**, the maximum amplitude of non-isothermal flow in the whole process is in nanoscale, while the scale of the acoustic streaming around the cluster is in microscale. It indicates that the non-isothermal flow is not a key factor to drive the particles away from the center.

References

[1] J. A. Sioss, C. D. Keating, *Nano Lett.* **2005**, *5*, 1779.

[2] A. Kuijk, A. van Blaaderen, A. Imhof, *J. Am. Chem. Soc.* **2011**, *133*, 2346.

[3] P. B. Muller, H. Bruus, *Phys. Rev. E* **2014**, *90*, 043016.

[4] M. Ohlin, PhD thesis, Ultrasonic Fluid and Cell Manipulation. *KTH Royal Institute of Technology*, **2015**.

[5] M. B. Moffett, *Science* **1970**, *170*, 156.

[6] A. D. Pierce, Acoustics: an introduction to its physical principles and applications, *McGraw-Hill New York*, **1981**

[7] Blackstock, D. T., Fundamentals of physical acoustics, *John Wiley & Sons*, **2000**.

[8] P. B. Muller, R. Barnkob, M. J. H. Jensen, H. Bruus, *Lab on a Chip* **2012**, *12*, 4617.

[9] L. D. Landau, E. M. Lifshitz, Statistical Physics: V. 5: Course of Theoretical Physics, *Pergamon press*, **1969**.

[10] W. L. Nyborg, *J. Acoust. Soc. Am.* **1953**, *25*, 68.

[11] P. H. Jones, O. M. Maragò, G. Volpe, Optical tweezers: Principles and applications, *Cambridge University Press*, **2015**.
